# Blocking P2X7 receptor with AZ 10606120 exacerbates vascular hyperpermeability and inflammation in murine polymicrobial sepsis

**DOI:** 10.14814/phy2.15290

**Published:** 2022-06-06

**Authors:** Jamie E. Meegan, Padmini Komalavilas, Joyce Cheung‐Flynn, Tsz Wing Yim, Nathan D. Putz, Jordan J. Jesse, Kyle D. Smith, Tatiana N. Sidorova, Han Noo Ri Lee, Toria Tomasek, Ciara M. Shaver, Lorraine B. Ware, Colleen M. Brophy, Julie A. Bastarache

**Affiliations:** ^1^ 12328 Division of Allergy, Pulmonary and Critical Care Medicine Department of Medicine Vanderbilt University Medical Center Nashville Tennessee USA; ^2^ 12328 Division of Vascular Surgery Vanderbilt University Medical Center Nashville Tennessee USA; ^3^ 12328 Department of Pathology, Microbiology and Immunology Vanderbilt University Medical Center Nashville Tennessee USA; ^4^ 12328 Department of Cell and Developmental Biology Vanderbilt University Medical Center Nashville Tennessee USA

**Keywords:** AZ 10606120, inflammation, P2X7R, sepsis, vascular dysfunction

## Abstract

Sepsis is a devastating disease with high morbidity and mortality and no specific treatments. The pathophysiology of sepsis involves a hyperinflammatory response and release of damage‐associated molecular patterns (DAMPs), including adenosine triphosphate (ATP), from activated and dying cells. Purinergic receptors activated by ATP have gained attention for their roles in sepsis, which can be pro‐ or anti‐inflammatory depending on the context. Current data regarding the role of ATP‐specific purinergic receptor P2X7 (P2X7R) in vascular function and inflammation during sepsis are conflicting, and its role on the endothelium has not been well characterized. In this study, we hypothesized that the P2X7R antagonist AZ 10606120 (AZ106) would prevent endothelial dysfunction during sepsis. As proof of concept, we first demonstrated the ability of AZ106 (10 µM) to prevent endothelial dysfunction in intact rat aorta in response to IL‐1β, an inflammatory mediator upregulated during sepsis. Likewise, blocking P2X7R with AZ106 (10 µg/g) reduced the impairment of endothelial‐dependent relaxation in mice subjected to intraperitoneal injection of cecal slurry (CS), a model of polymicrobial sepsis. However, contrary to our hypothesis, AZ106 did not improve microvascular permeability or injury, lung apoptosis, or illness severity in mice subjected to CS. Instead, AZ106 elevated spleen bacterial burden and circulating inflammatory markers. In conclusion, antagonism of P2X7R signaling during sepsis appears to disrupt the balance between its roles in inflammatory, antimicrobial, and vascular function.

## Graphical abstract

In this study, blocking the P2X7R with the specific and highly selective antagonist AZ 10606120 completely restored vascular dysfunction in rat aorta in response to IL‐1β and partially restored vascular dysfunction in a murine model of polymicrobial sepsis induced by intraperitoneal injection of cecal slurry. However, AZ106 did not confer protective benefits regarding vascular hyperpermeability or injury, lung apoptosis, circulating inflammatory markers, illness severity, or bacterial burden. Conversely, AZ106 treatment in the CS model resulted in increased spleen bacterial burden and circulating inflammatory markers.

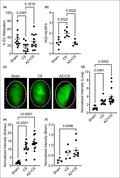

## 1 INTRODUCTION

Sepsis, a “life‐threatening organ dysfunction caused by a dysregulated host response to infection” (Singer et al., 2016), is a major burden worldwide with high morbidity and mortality and no specific therapy to prevent or treat the disease process. Although decades of research have investigated the pathophysiology of sepsis, the complexity of the disease and the presence of major comorbidities have limited clinical care to treating an infection with antibiotics and supporting organ function with fluids, vasopressors, and oxygen. The endothelium that lines the blood vessel lumen plays a central role in regulating physiological homeostasis of blood flow, tissue delivery of oxygen and nutrients, and the inflammatory response. Endothelial function becomes disrupted during sepsis, leading to dysregulated blood flow and increased microvascular permeability, which contribute to tissue edema, organ dysfunction, and ultimately death (Ince et al., 2016). Many therapies showing great promise in pre‐clinical models, including those targeting inflammatory mediators such as lipopolysaccharide (LPS) (Ziegler et al., 1991) and tumor necrosis factor‐α (TNF‐α) (Reinhart & Karzai, 2001), coagulation pathways, and oxidative injury, have failed to show a survival benefit in clinical trials (Marshall, 2014). Therefore, there is a need to uncover specific mechanisms involved in the dysregulated vascular response during sepsis so that more targeted therapies can be developed.

Purinergic receptors on the endothelium are critical for the regulation of the cellular response to extracellular signaling molecules such as adenosine triphosphate (ATP) and other inflammatory mediators that are increased during sepsis due to cellular stress and increased pro‐inflammatory signaling. The P2X7 receptor (P2X7R), which primarily acts via NLRP3 inflammasome‐mediated signaling, has been shown to be critical for bacterial killing in monocytes and macrophages (Csoka et al., 2015; Hill et al., 2010), but the role of P2X7R on the endothelium during sepsis has not been well characterized. Our group previously showed that activation of P2X7R leads to impaired endothelial‐dependent relaxation in response to stretch injury (Guth et al., 2017; Komalavilas et al., 2017; Luo et al., 2016, 2017), exogenous ATP stimulation (Guth et al., 2017; Komalavilas et al., 2017), and acidic normal saline solutions (Cheung‐Flynn et al., 2019). Additionally, other groups have identified a protective role for P2X7R antagonism in several experimental models of sepsis (Arulkumaran et al., 2018; Santana et al., 2015; Savio et al., 2017; Wu et al., 2017). In a cecal ligation and puncture (CLP) model, genetic knockout of P2X7R protected mice against inflammation, lung injury, and mortality (Santana et al., 2015), as well as brain oxidative stress (Savio et al., 2017). Additionally, pharmacological blockade of P2X7R reduced inflammatory cytokines and intestinal barrier disruption during CLP‐induced sepsis (Wu et al., 2017), and reduced renal injury during a fecal peritonitis model of sepsis. However, the role of P2X7R in modulating vascular function during sepsis has yet to be elucidated. Therefore, we hypothesized that the P2X7R antagonist AZ 10606120 (AZ106) would prevent vascular dysfunction and subsequent negative outcomes in a murine polymicrobial cecal slurry (CS) experimental model of sepsis.

## 2 MATERIALS AND METHODS

### 2.1 Animals

This study was carried out in strict accordance with the recommendations in the Guide for the Care and Use of Laboratory Animals of the National Institutes of Health. All animal experiments were approved by the Vanderbilt Institutional Animal Care and Use Committee.

### 2.2 Measurement of endothelial‐dependent relaxation

To determine the effect of AZ106 on endothelial dysfunction, rat aortae were collected from euthanized female Sprague Dawley rats (250–300 g). Immediately after euthanasia by CO_2_ exposure, the aorta was isolated and cut into rings (1–2 mm) that were suspended in a muscle bath containing a bicarbonate buffer (120 mM sodium chloride, 4.7 mM potassium chloride (KCl), 1.0 mM magnesium sulfate, 1.0 mM monosodium phosphate, 10 mM glucose, 1.5 mM calcium chloride, and 25 mM sodium bicarbonate, pH 7.4) equilibrated with 95% O_2_/5% CO_2_ at 37°C for 1 h at a resting tension of 1 g. The tissues were then manually stretched to three times the resting tension and maintained at resting tension for an additional 1 h to produce the maximal force tension relationship as previously described (Cheung‐Flynn et al., 2019). After equilibration, the rings were primed with 110 mM KCl (with equimolar replacement of sodium chloride in bicarbonate buffer) to determine functional viability. Viable rings were incubated in buffer or IL‐1β (50 ng/ml) in the presence or absence of AZ106 (10 µM, 1‐h pre‐treatment) for 2 h. Tissues were tested for contractile response to a dose of phenylephrine (PE) to yield submaximal contraction (approximately 60%–70% of maximum KCl; 1–5 × 10^−7^ M) and relaxed with carbachol (CCH, 10^−8^ to 10^−5^ M), an acetylcholine analog, to determine endothelial‐dependent relaxation responses. Force measurements were obtained using the Radnoti force transducer (model 159901A; Radnoti LLC) interfaced with a PowerLab data acquisition system and Chart software (AD Instruments Inc., Colorado Springs, CO) and converted to stress by adjusting to the length and weight of the tissue. Percent relaxation was calculated as a change in stress compared to the maximal PE‐induced contraction which was set as 100%. Endothelial relaxation of aortae isolated from mice pre‐treated for 1 h with AZ106 (10 µg/g, SQ) before being subjected to control or cecal slurry (CS) for 24 h was measured using a modification of the above methodology as described previously (Meegan et al., 2020).

### 2.3 Cecal slurry polymicrobial sepsis model

The cecal slurry peritonitis model was adapted from a neonatal necrotizing enterocolitis model described previously (Wynn et al., 2007, 2008). Cecal slurry (CS) was prepared from 6‐week‐old female C57BL/6 mice purchased from The Jackson Laboratory (Bar Harbor, ME) by isolating cecal contents from euthanized donors as previously described (Meegan et al., 2020; Shaver et al., 2019). Intraperitoneal injection of CS at 1.7−2.0 mg/g body weight or 5% dextrose control was given to 8–12‐week‐old male C57BL/6 mice to induce polymicrobial peritoneal sepsis in the presence or absence of a single 1‐h pre‐treatment dose of P2X7R antagonist AZ 10606120 (AZ106; 10 µg/g body weight, SQ). The dose of AZ106 chosen for these studies was based on reports in the literature (Giannuzzo et al., 2016; Wu et al., 2017; Yan et al., 2015) and we confirmed adequate P2X7R signaling blockade through lack of induction of downstream NLRP3 transcription. Whole lungs were excised, flash frozen, and stored at −80°C. mRNA was extracted from whole lungs using Qiagen RNeasy Plus Mini Kit (Hilden, Germany). cDNA was generated using a SuperScript VILO cDNA Synthesis Kit (Invitrogen, Carlsbad, CA). NLRP3 was quantified by qPCR and normalized to GAPDH expression using TaqMan primer probes (Thermo Fisher Scientific). After 6 and 24 h, mice were evaluated for sepsis severity using a validated scoring method (Manley et al., 2005; Su et al., 2013). Specifically, clinical severity markers were determined by researchers blinded to treatment groups using the following criteria to formulate a composite sepsis severity score: (A) Response to finger poke (4 = normal response, 3 = decreased response, 2 = severely decreased response, 1 = minimal response, 0 = no response (deceased)), (B) signs of encephalopathy (4 = normal, 3 = tremors, staggering, 2 = twisting, 1 = turning and flipping, 0 = no response (deceased)), (C) appearance (4 = normal, with one point subtracted for any of the following: Piloerection, periorbital exudates, respiratory distress, diarrhea). Bacterial counts of the lung and spleen were measured as previously described (Meegan et al., 2020). Aortae were collected for the measurement of endothelial‐dependent relaxation (described above).

### 2.4 Vascular permeability

After 24 h of CS with or without AZ106 pre‐treatment (10 µg/g, SQ), vascular permeability in the lung, kidney, and brain was measured directly by retroorbital injection of the 70 kDa fluorescent probe Angiosense^®^ 750EX (2 nM, 100 μl; Perkin Elmer) as previously described (Kerchberger et al., 2019; Meegan et al., 2020). Accumulation of Angiosense^®^ 750EX in excised tissue was measured using a Li‐Cor Pearl camera.

### 2.5 Measure of vascular injury markers

Vascular injury markers were measured in duplicate in plasma on the Milliplex Mouse Cardiovascular Disease (CVD) Magnetic Bead Panel‐1 (Millipore‐Sigma) per manufacturer's instructions.

### 2.6 Detection of apoptosis in lung endothelium: TUNEL and MECA‐32 immunofluorescence

Twenty‐four hours after CS, lungs were collected and formalin‐fixed. Five‐µm thick paraffin sections were cut, deparaffinized and rehydrated using descending alcohol series. Antigen retrieval was performed in 10 mM sodium citrate with 0.05% Tween‐20 (pH 6.0) at 95°C (water bath) for 10 min. After washing with PBS, terminal deoxynucleotidyl transferase dUTP nick end labeling (TUNEL) assay was performed with In Situ Cell Death Detection Kit, Fluorescein (Sigma Aldrich). Slides were incubated in the dark with TUNEL reaction mixture at 37°C for 1 h. Slides were rinsed three times with PBS, blocked with 10% normal goat serum in PBS and 0.05% Tween‐20 for 1 h, and incubated with rat anti‐mouse monoclonal MECA‐32 (panendothelial) antibody (1:100, BioxCell BE0200) in PBS, 3% normal goat serum, 0.05% Tween‐20 overnight at 4°C for endothelial labeling. Following the primary antibody, slides were rinsed with PBS for 5 min three times and incubated with goat anti‐rat Alexa Fluor 555‐conjugated secondary antibody (A21434, Life Technology), diluted 1:500 in PBS with 1% normal goat serum and 0.05% Tween‐20. After washing with PBS, all sections were counterstained by applying cover slips with ProLong Gold antifade reagent with DAPI. Negative control staining was also performed on separate sections with rat IgG substituted for primary antibody. Within 24 h, immunofluorescence‐stained sections were examined and photographed under confocal microscopy (LSM880 Airy Scan, Zeiss) by a researcher blinded to the treatment group. Five low power (10X) and 10 high power (60X) non‐overlapping visual fields were randomly selected for each section. Images were quantified by counting the number of cells with TUNEL‐positive staining per field by a researcher blinded to treatment group.

### 2.7 Measure of inflammatory cytokines

Inflammatory cytokines were measured in duplicate in plasma using the Mouse Proinflammatory Panel‐1 from Meso Scale Discovery Multi‐Plex Biomarker Assay (Meso Scale Diagnostics, LLC, Rockville, MD) per manufacturer's instructions.

### 2.8 Statistical analysis

Statistical analysis was performed using GraphPad Prism (8.3.0). One‐way ANOVA with Tukey's multiple comparisons post hoc test was performed to analyze differences between groups with *α* = 0.05. Graphs are represented as mean ± SEM.

## 3 RESULTS

### 3.1 Blocking P2X7 receptor with AZ 10606120 prevents vascular dysfunction in rat aorta induced by IL‐1β

**FIGURE 1:**
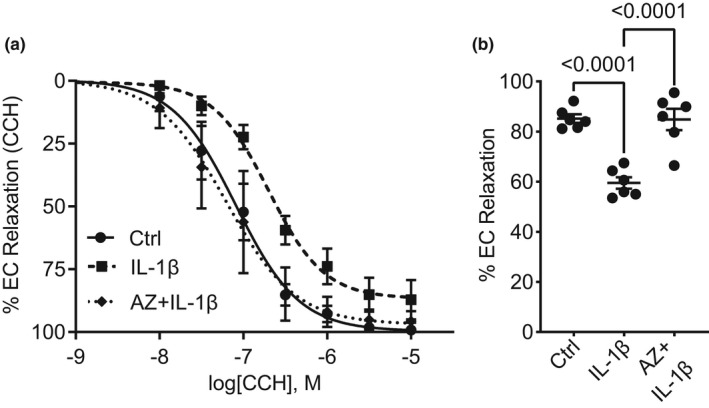
Blocking P2X7 receptor with AZ 10606120 prevents vascular dysfunction in rat aorta induced by IL‐1β. Freshly isolated rat aortae were subjected to buffer (Ctrl) or IL‐1β (50 ng/ml) in the presence or absence of AZ 10606120 (AZ; 10 µM) for 2 h. (a) Rat aortae were suspended in a muscle bath, contracted with PE, and treated with escalating doses of carbachol (CCH; 10^−8^–10^−5^ M). The percent relaxation was determined as a change to the maximal PE‐induced contraction. (b) Percent relaxation to 5 × 10^−7^ M CCH shows AZ 10606120 prevents dysfunction of endothelial‐dependent relaxation induced by IL‐1β (*n* = 6). Statistical analysis was performed using one‐way ANOVA with Tukey's multiple comparisons. Graphs represent mean ± SEM

Our previous study demonstrated that the pro‐inflammatory cytokine IL‐1β disrupted endothelial‐dependent relaxation (Cheung‐Flynn et al., 2019). Since IL‐1β is known to be elevated during sepsis, we tested the ability of P2X7R antagonist AZ106 to prevent vascular dysfunction in response to IL‐1β as proof of concept. Indeed, AZ106 (10 µM) attenuated the reduction of endothelial‐dependent relaxation in rat aortae in response to IL‐1β (50 ng/ml), indicating a potential role for endothelial P2X7R during inflammatory conditions (Figure 1).

### 3.2 Blocking P2X7 receptor with AZ 10606120 partially prevents vascular dysfunction, but not hyperpermeability, during murine sepsis

**FIGURE 2:**
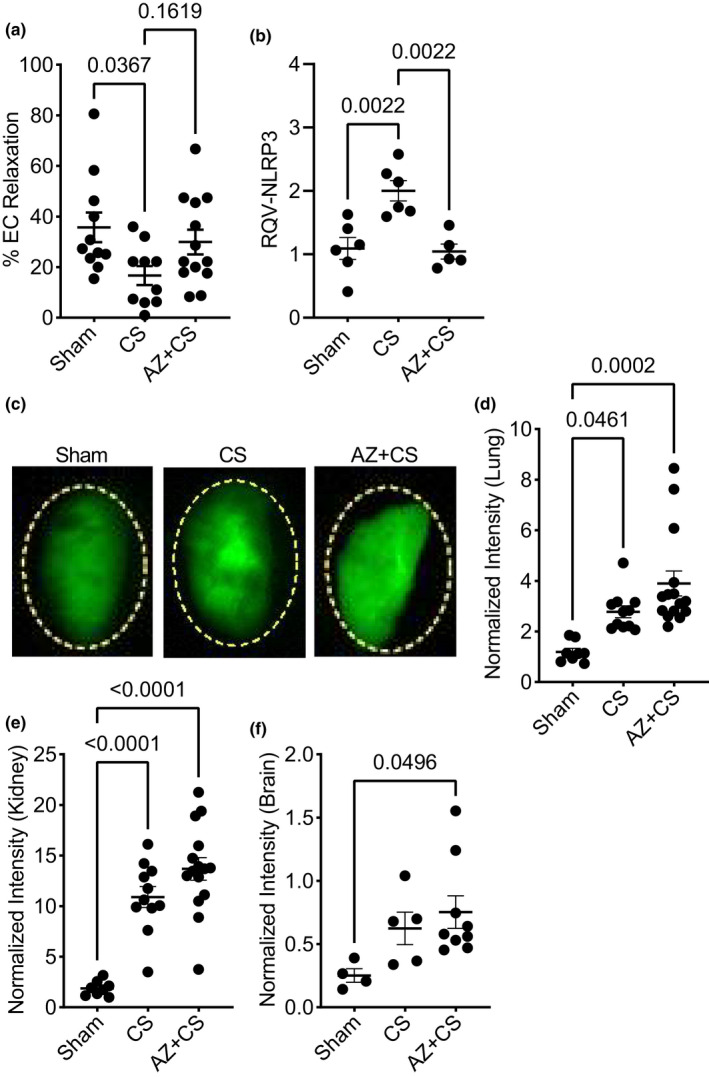
Blocking P2X7 receptor with AZ 10606120 partially prevents vascular dysfunction, but not permeability, during murine sepsis. Mice were subjected to cecal slurry (CS) to induce sepsis in the presence or absence of 1‐h pre‐treatment with AZ 10606120 (AZ; 10 µg/g, SQ) for 24 h. (a) Endothelial‐dependent relaxation of mouse aortae (percent relaxation to 5 × 10^−7^ M CCH) decreased in mice subjected to CS; pre‐treatment with AZ 10606120 was not statistically different from control (sham) mice (*n* = 10–13). (b) Blocking P2X7R with this dose of AZ 10606120 was sufficient to prevent the downstream increase in NLRP3 transcription in response to CS in lung tissue. (c) Representative fluorescence images and (d) quantification of Angiosense accumulation in the lungs shows pre‐treatment with AZ 10606120 in mice subjected to CS did not prevent vascular hyperpermeability (*n* = 8–15). Likewise, AZ 10606120 did not prevent vascular hyperpermeability in (e) kidney (*n* = 8–15) or (f) brain (*n* = 4–9) of mice subjected to CS. Statistical analysis was performed using one‐way ANOVA with Tukey's multiple comparisons. Graphs represent mean ± SEM

Due to our initial observations of the ability of AZ106 to prevent endothelial dysfunction in response to IL‐1β, we hypothesized that AZ106 might prevent endothelial dysfunction during murine polymicrobial sepsis. Therefore, we tested pre‐treatment (1 h) of AZ106 (10 µg/g) in a cecal slurry (CS) polymicrobial model of sepsis in mice. Aortae isolated from mice subjected to CS displayed a significant reduction in endothelial‐dependent relaxation (Figure 2a). AZ106 partially reduced the vascular dysfunction induced by CS (*p* = 0.16). Treatment with AZ106 also prevented the increase in NLRP3 transcription in response to CS in lung tissue (Figure 2b). In addition to regulating vascular tone, the endothelium plays an important role in maintaining a tight vascular barrier. During inflammatory conditions like sepsis, this barrier is compromised, leading to fluid accumulation in the tissue and limiting the supply of essential oxygen and nutrients necessary to maintain homeostasis. Next, we sought to determine if AZ106 could protect vascular barrier integrity. To evaluate vascular hyperpermeability, we measured tissue accumulation of a 70 kDa fluorescent protein, Angiosense^®^, in several organs affected by sepsis. However, contrary to our hypothesis, AZ106 did not prevent vascular hyperpermeability during sepsis in the lung (Figure 2c–d), kidney (Figure 2e), or brain (Figure 2f).

### 3.3 Blocking P2X7 receptor with AZ 10606120 does not prevent vascular injury during murine sepsis

**FIGURE 3:**
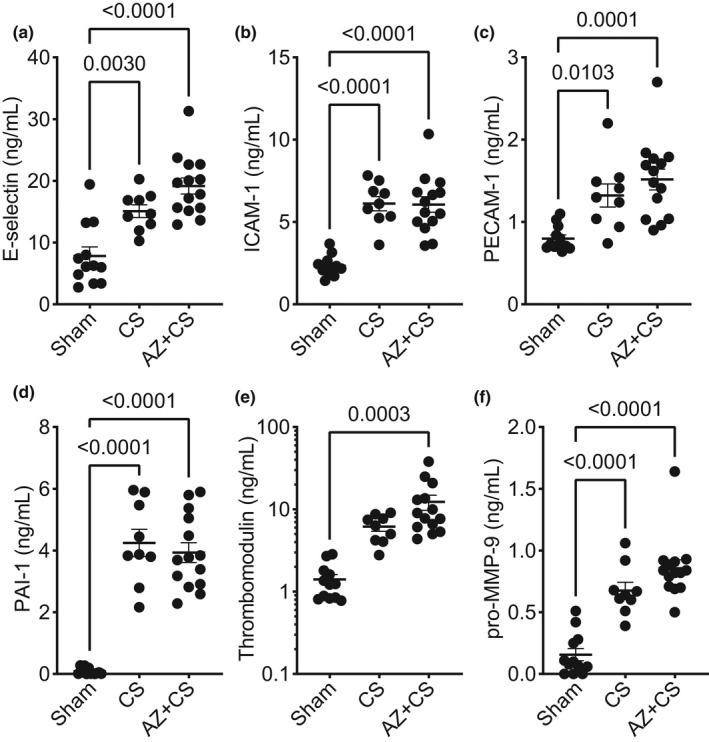
Blocking P2X7 receptor with AZ 10606120 does not prevent vascular injury during murine sepsis. Mice were subjected to cecal slurry (CS) to induce sepsis in the presence or absence of 1‐h pre‐treatment with AZ 10606120 (AZ; 10 µg/g, SQ) for 24 h. Circulating markers of vascular injury (plasma), including (a) E‐selectin, (b) ICAM‐1, (c) PECAM‐1, (d) PAI‐1, (e) thrombomodulin, or (f) pro‐MMP‐9, were not reduced with AZ 10606120 treatment in mice subjected to CS. Statistical analysis was performed using one‐way ANOVA with Tukey's multiple comparisons. Graphs represent mean ± SEM (*n* = 9–14)

In addition to vascular dysfunction and hyperpermeability, we evaluated the circulating levels of several vascular injury markers in mice during sepsis. AZ106 did not prevent increased levels of circulating E‐selectin (Figure 3a), ICAM‐1 (Figure 3b), PECAM‐1 (Figure 3c), PAI‐1 (Figure 3d), thrombomodulin (Figure 3e), or pro‐MMP‐9 (Figure 3f) in mice subjected to CS.

### 3.4 Blocking P2X7 receptor with AZ 10606120 does not prevent lung apoptosis during murine sepsis

**FIGURE 4:**
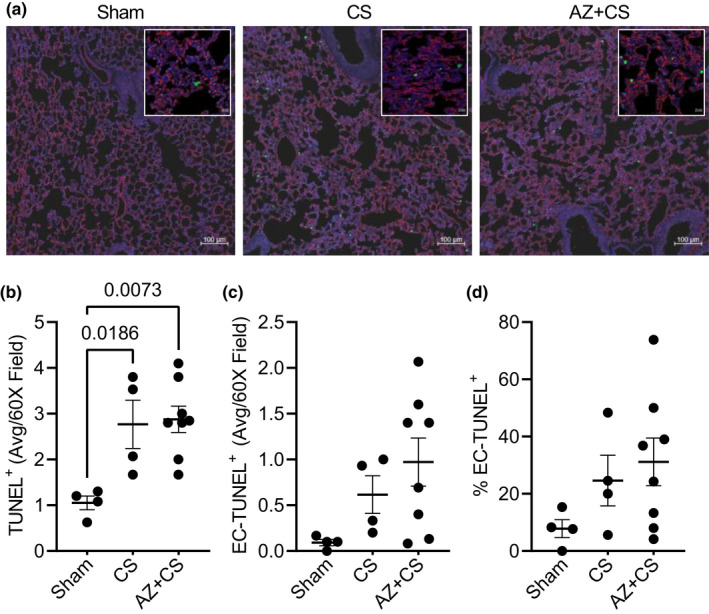
Blocking P2X7 receptor with AZ 10606120 does not prevent lung apoptosis during murine sepsis. Mice were subjected to cecal slurry (CS) to induce sepsis in the presence or absence of 1‐h pre‐treatment with AZ 10606120 (AZ; 10 µg/g, SQ) for 24 h. (a) Representative images of apoptotic (TUNEL) fluorescence staining are shown at 10X and 60X (insert) power [blue = DAPI (nuclei), red = MECA‐32 (endothelial cells), green = TUNEL (apoptotic cells)]. (b) Quantification of TUNEL‐positive fluorescence staining shows AZ 10606120 did not prevent apoptosis in lung tissue of mice subjected to CS. There were no statistical differences in the (c) number or (d) percentage of TUNEL‐positive cells that were endothelial (overlap of MECA‐32 [red] on TUNEL [green]). Statistical analysis was performed using one‐way ANOVA with Tukey's multiple comparisons. Graphs represent mean ± SEM (*n* = 4–8)

Apoptosis of multiple cell types occurs during sepsis and contributes to vascular and organ dysfunction. Studies from our group and others show that apoptosis of lung endothelium contributes to sepsis‐induced lung injury (Gill et al., 2015; Meegan et al., 2020). Therefore, we evaluated apoptosis using TUNEL staining in the lungs of mice subjected to sepsis. Though the number of apoptotic cells in lung tissue increased in mice subjected to CS, AZ106 did not prevent apoptosis (Figure 4a,b). Neither the total number (Figure 4c) nor percentage (Figure 4d) of TUNEL‐positive cells that were endothelial cells differed between groups.

### 3.5 Blocking P2X7 receptor with AZ 10606120 does not prevent increased circulating inflammatory markers during murine sepsis

**FIGURE 5:**
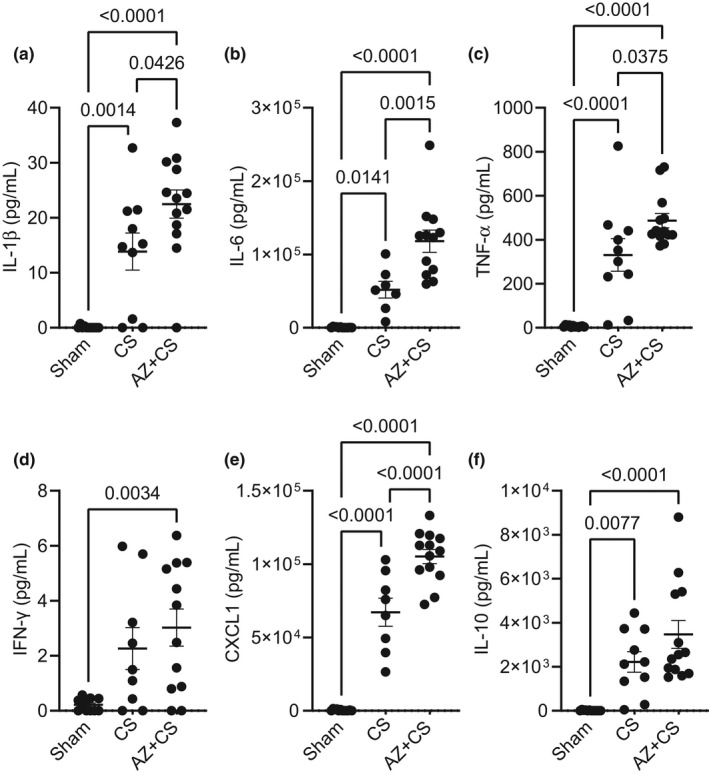
Blocking P2X7 receptor with AZ 10606120 does not prevent increased circulating inflammatory markers during murine sepsis. Mice were subjected to cecal slurry (CS) to induce sepsis in the presence or absence of 1‐h pre‐treatment with AZ 10606120 (AZ; 10 µg/g, SQ) for 24 h. Circulating inflammatory markers (plasma), including (a) IL‐1β, (b) IL‐6, (c) TNF‐α, (d) IFN‐γ, (e) CXCL1, or (f) IL‐10, were not reduced with AZ 10606120 treatment in mice subjected to CS. Moreover, several levels of inflammatory markers (IL‐1β, IL‐6, TNF‐α, CXCL1) were worsened with AZ 10606120 treatment. Statistical analysis was performed using one‐way ANOVA with Tukey's multiple comparisons. Graphs represent mean ± SEM (*n* = 7–13)

To identify a possible explanation for the inability of AZ106 to prevent vascular hyperpermeability, vascular injury, or lung apoptosis, we evaluated the circulating levels of several inflammatory markers in mice during sepsis. AZ106 did not prevent the increased levels of circulating IL‐1β (Figure 5a), IL‐6 (Figure 5b), TNF‐α (Figure 5c), IFN‐γ (Figure 5d), CXCL1 (Figure 5e), or IL‐10 (Figure 5f) in mice subjected to CS. Conversely, several of these cytokines (IL‐1β, IL‐6, TNF‐α, CXCL1) were significantly elevated in AZ106‐treated mice compared to those subjected to CS alone, indicating a potential role for P2X7R in the modulation of the inflammatory response during sepsis.

### 3.6 Blocking P2X7 receptor with AZ 10606120 does not reduce illness severity or bacterial load during murine sepsis

**FIGURE 6:**
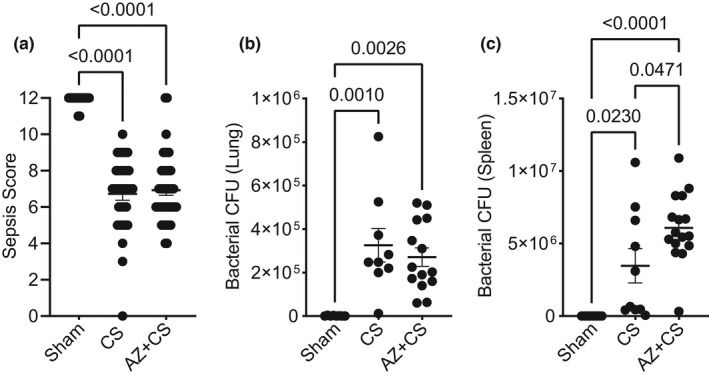
Blocking P2X7 receptor with AZ 10606120 does not prevent illness severity or bacterial load during murine sepsis. Mice were subjected to cecal slurry (CS) to induce sepsis in the presence or absence of 1‐h pre‐treatment with AZ 10606120 (AZ; 10 µg/g, SQ) for 24 h. AZ 10606120 treatment did not reduce (a) sepsis illness severity (sepsis score, *n* = 30–42) or bacterial load (CFU) of the (b) lung (*n* = 8–16) or (d) spleen (*n* = 8–16). Conversely, spleen bacterial load worsened with AZ 10606120 treatment. Statistical analysis was performed using one‐way ANOVA with Tukey's multiple comparisons. Graphs represent mean ± SEM

During sepsis, vascular dysfunction and inflammation are partially driven by inadequate control of infection. Thus, we evaluated overall sepsis disease severity (sepsis score) and bacterial burden in our CS experimental model. While sepsis severity score worsened (Figure 6a) and bacterial burden in the lung (Figure 6b) and spleen (Figure 6c) were greatly elevated with CS, there was no improvement with AZ106 treatment. In fact, spleen bacterial burden was further elevated in the AZ106 group, suggesting a potentially important antimicrobial role for P2X7R that is inhibited by AZ106.

## 4 DISCUSSION

In this study, blocking the P2X7R with the specific and highly selective antagonist AZ 10606120 completely restored vascular dysfunction in rat aorta in response to IL‐1β and partially restored vascular dysfunction in a murine model of polymicrobial sepsis induced by intraperitoneal injection of cecal slurry. However, AZ106 did not confer protective benefits regarding vascular hyperpermeability or injury, lung apoptosis, circulating inflammatory markers, illness severity, or bacterial burden. Conversely, AZ106 treatment in the CS model resulted in increased spleen bacterial burden and increased circulating inflammatory markers. Of note, only one animal in the CS group did not survive during this study. As in our previous studies (Bastarache et al., 2021; Meegan et al., 2020), mortality is not increased at 24 h in this model; therefore, a limitation of this study is that it does not address the effects of AZ106 administration on mortality, an important area for future study.

Though several groups have shown benefits of P2X7R blockade in sepsis using P2X7R null mice in the cecal ligation and puncture (CLP) model (Santana et al., 2015; Savio et al., 2017), or P2X7R antagonists in a mouse CLP model (Wu et al., 2017) or rat fecal slurry model (Arulkumaran et al., 2018), our mouse model of cecal slurry‐induced sepsis and the use of a different antagonist compound may have contributed to the differences in our results of P2X7R antagonism compared to other reports. We chose to test the AZ106 compound in the CS model of sepsis for several reasons. First, injections of CS are more consistent and reproducible than surgical models such as CLP and avoid the need for survival surgery. CS injections can also be titrated to induce desired illness severity, and the CS model produces organ‐specific vascular hyperpermeability and clinically relevant end‐organ dysfunction, as we and others have previously demonstrated (Bastarache et al., 2021; Meegan et al., 2020; Wynn et al., 2007, 2008). Regarding the use of AZ106 over other P2X7R inhibition strategies, we opted for a pharmacological antagonist over a knockout mouse model to enhance the potential for therapeutic translatability. We chose AZ106, a negative allosteric modulator, over the competitive antagonists A740003 or A438079, as non‐competitive antagonists are generally safer and may have higher therapeutic potential. However, it may be possible that the function of AZ106 as a non‐competitive antagonist could become overwhelmed during hyperinflammatory conditions such as sepsis. On the other hand, others have reported a necessary and protective role for P2X7R expression, likely due to its high expression on immune cells, including monocytes, macrophages, and neutrophils (Csoka et al., 2015; Greve et al., 2017; Jacob et al., 2013; Morandini et al., 2014). Our finding that P2X7R antagonism prevented large vessel vascular dysfunction but not microvascular hyperpermeability or injury, lung apoptosis, inflammatory markers, illness severity, or bacterial burden during sepsis *in vivo* could indicate that organ‐ or cell‐specific rather than global targeting of the receptor may be required to improve outcomes. Indeed, our finding that spleen bacterial counts and circulating inflammatory markers were elevated with AZ106 treatment lends evidence to the hypothesis that P2X7R on inflammatory cells is necessary for bacterial clearance and killing. The blockade of P2X7R on these immune cells leading to increased bacterial burden could therefore contribute to continued immune cell activation and subsequent exacerbation of inflammation. Perhaps endothelial‐specific targeting of P2X7R without affecting its function on inflammatory cells would produce more promising results.

We also acknowledge that responses seen with sections of intact tissue can vastly differ from those in a complex, whole organism. Assessment of P2X7R activation in vivo is challenging as downstream signaling is not specific and thus is difficult to interpret. AZ106 administration blocked the transcriptional upregulation of P2X7R's most prominent downstream effector, NLRP3, during CS in lung tissue even after 24 h. However, we also observed increased circulating inflammatory markers such as IL‐1β. This may indicate that modulation of P2X7R among different tissue beds and cell types in an inflammatory context results in different downstream effects (i.e., perhaps the increased circulating IL1‐β arises from outside the lung). Furthermore, macrovascular (relaxation) and microvascular (permeability) endothelial responses are distinct and may operate via different pathways. Hence, blocking P2X7R on these different endothelial cell types and in different tissue beds could result in distinct consequences. Another consideration for the lack of benefit of AZ106 in our sepsis model is the timing of administration. As P2X7R activation and NLRP3 modulation may be required for the initial inflammatory response, perhaps administering AZ106 later (during the resolution phase) would provide more benefit. In fact, studies showing a benefit of P2X7R antagonists during sepsis, including A740003 in a mouse CLP model (Wu et al., 2017) and A438079 in a rat fecal slurry model (Arulkumaran et al., 2018), administered the antagonists 24 and 2 h, respectively, after sepsis injury induction. Going forward, it will be important to determine the role of P2X7R in individual cell types at each phase of disease, especially during complex, systemic, pathophysiological processes like sepsis

Another possible explanation for the lack of protection with P2X7R blockade is the presence of other purinergic receptors. The P2X receptor family has seven subtypes, and the high expression of P2X4 and P2X5 receptors on the endothelium (Ramirez & Kunze, 2002; Schwiebert et al., 2002) could indicate a significant role in the endothelial response to inflammatory mediators. Indeed, AZ106 is a highly potent and specific P2X7R antagonist with little effect on other P2X subtypes (Guile et al., 2009). Additionally, the P2X receptors interact with many intracellular signaling proteins, so targeting downstream pathways may be required to avoid off‐target effects (Müller et al., 2020). Furthermore, P2Y receptors have also been shown to play a role in regulating the endothelial response during inflammatory injury (Battistone et al., 2020; Zemskov et al., 2011). It will be important in future studies to investigate the contribution of each of these receptors in different pathological contexts.

Although the systemic administration of the P2X7R antagonist AZ106 failed to protect mice against a cecal slurry peritonitis experimental model of sepsis, the results of our study suggest that P2X7R activation plays distinct roles depending on context: The role of P2X7R in modulating vascular function seems to depend on its location in the *macro*vascular versus *micro*vascular endothelium, and its expression on inflammatory cells may play an important role in modulating the inflammatory response. Considering this, future studies delineating these differences are warranted for potential development of P2X7R targeted therapeutics in sepsis.

## CONFLICT OF INTEREST

The authors declare no competing interests.

## AUTHOR CONTRIBUTIONS

This study was conceived and coordinated by J.E.M., P.K., J.C., C.M.B., and J.A.B. P.K., J.C., T.W.Y., N.D.P., J.J.J., K.D.S., T.N.S, H.N.R.L., and T.T. performed experiments. J.E.M. and P.K. analyzed data with input from C.M.B. and J.A.B. Data was interpreted by J.E.M., P.K., J.C., C.M.S., L.B.W., C.M.B., and J.A.B. J.E.M. created the figures and wrote the manuscript. All authors critically revised and approved the manuscript.

## ETHICS STATEMENT

This study was carried out in strict accordance with the recommendations in the Guide for the Care and Use of Laboratory Animals of the National Institutes of Health. All animal experiments were approved by the Vanderbilt Institutional Animal Care and Use Committee.
